# The Painless Removal of Pulps by the Use of Chloretone

**Published:** 1905-02-15

**Authors:** D. W. Babcock


### THE PAINLESS REMOVAL OF PULPS BY THE USE OF CHLORETONE.

BY D. W. BABCOCK, D.D.S.

I do not claim to be the originator of the idea of the use of Chloretone for local anesthesia in my profession only so far as the application. I first saw an account, in the *American Medical Journal*, where it had been successfully used for a minor operation and I came to the conclusion if it could be used for that purpose, I could make use of it in my practice. The article gave it used as a saturated solution of chloretone with ether.

In the removal of pulps in "simple exposure" or "superficial pulpitis," I find it par excellence and will explain my method:

Apply the rubber dam, fill the hypodermic syringe and force into the pulp the saturated solution of chloretone and ether. When the exposure is large, I take some of the crystals and by pressure, anesthetize the pulp at the exposure, then by using a piece of vulcanite rubber and holding it securely around the needle, succeed in doing the operation without pain. It must be forced into the pulp under pressure otherwise one might as well use aqua pura. After using the solution hypodermatically, one can then use the broach and remove the pulp absolutely without pain, and after the hemorrhage has stopped, immediately proceed to fill the root canals and complete the filling at the same sitting. I have used it the past four years and have yet to have a failure.

It is necessary, in using it for distal and occlusal cavities in posterior teeth, to have curved needles which are easily procured at the manufacturers; the straight needle answering in all other positions. Chloretone having antiseptic properties certainly causes no injury to the root canals.

The objections to its use are: It is painful to apply, but with a little care on the part of the operator it will cause no more pain than the application of cocain or arsenious oxide; it produces quite a hemorrhage, seldom lasting more than fifteen minutes, and when we compare this with the time that it takes in using arsenious oxide, it is quite a saving to the busy practitioner. The best results are obtained by preparing the solution at the time of using, as it seems to deteriorate by standing in solution, consequently I keep the chloretone and ether in my medicine case, mixing them when necessary to use.

I wish all practitioners would try it. I feel satisfied that they will use cocain, and that very painful devitalizer arsenious oxide no more.

